# Alginate oligosaccharide enhances intestinal integrity of weaned pigs through altering intestinal inflammatory responses and antioxidant status

**DOI:** 10.1039/c8ra01943f

**Published:** 2018-04-10

**Authors:** Jin Wan, Jiao Zhang, Daiwen Chen, Bing Yu, Zhiqing Huang, Xiangbing Mao, Ping Zheng, Jie Yu, Jun He

**Affiliations:** Institute of Animal Nutrition, Sichuan Agricultural University Chengdu 611130 Sichuan People's Republic of China wanjin91@163.com hejun8067@163.com +86-28-86290920 +86-13-419354223

## Abstract

Alginate oligosaccharide (AOS), prepared from depolymerised alginate, a natural polysaccharide occurring in the cell walls of brown algae, provides beneficial effects for intestinal health. However, the underlying mechanisms by which AOS supplementation maintains the intestinal integrity of weaned pigs remain obscure. Here, we aimed to determine how AOS modulates the intestinal integrity of weaned pigs. Twenty-four weaned pigs were assigned to two treatments: a control group (basal diet) and an AOS group (the basal diet supplemented with 100 mg kg^−1^ AOS). On day 15, eight pigs per treatment were randomly selected and sacrificed for serum and intestinal samples. We observed that AOS supplementation enhanced the intestinal integrity, as evidenced by the increased (*P* < 0.05) intestinal occludin protein abundance. Compared to the control group, AOS ingestion both elevated (*P* < 0.05) the jejunal and ileal catalase activity and decreased (*P* < 0.05) the duodenal and jejunal tumour necrosis factor-α concentration and mast cell tryptase expression. Furthermore, AOS down-regulated (*P* < 0.05) the duodenal toll-like receptor 4 (TLR4) and its down-stream signals, myeloid differentiation factor 88 (MyD88), interleukin-1 receptor-associated kinase 1 (IRAK1) and tumour necrosis factor receptor-associated factor 6 (TRAF6) mRNA levels, as well as jejunal nucleotide-binding oligomerisation domain protein 1 (NOD1) and its adaptor molecule, receptor-interacting serine/threonine-protein kinase 2 (RIPK2), mRNA levels. Additionally, phospho-nuclear factor-κB (p-NF-κB) p65 protein abundance in the duodenum and jejunum was down-regulated (*P* < 0.05) following AOS supplementation. According to the above results, the enhanced intestinal integrity in AOS-supplemented pigs appears to be associated with the elevated antioxidant capacity and the reduced mast cell degranulation, as well as the inhibited pro-inflammatory cytokines production *via* inhibiting the TLR4/NF-κB and NOD1/NF-κB signalling pathways.

## Abstract 2

Alginate oligosaccharide (AOS), prepared from depolymerised alginate, a natural polysaccharide occurring in the cell walls of brown algae, provides beneficial effects for intestinal health.

## Graphical abstract

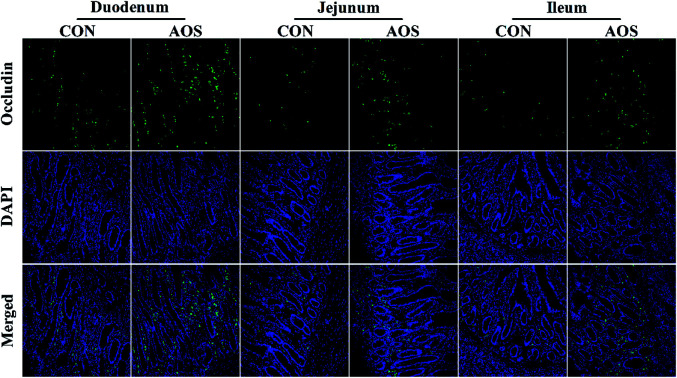

## Introduction

The intestinal barrier is a single layer of cells lining the gut that mainly consists of enterocyte membranes and tight junctions between enterocytes, and its integrity is essential for the digestion and absorption of nutrients, both in humans and animals.^1–3^ However, many factors, such as weaning, can induce impairment in the intestinal barrier integrity of piglets.^4–6^ Mast cell inflammatory mediator release (*e.g.*, proteases, histamine and cytokines) plays a major role in the intestinal barrier disruption during the post-weaning period.^7,8^ Hence, controlling the intestinal mast cell inflammatory mediators release may have benefits in alleviating weaning-induced intestinal barrier damage in piglets. Furthermore, weaning can disrupt the oxidative balance and cause oxidative injury in the small intestine of piglets, so maintaining the antioxidant capacity is also crucial in preserving intestinal integrity.^9,10^ For these reasons, various nutritional approaches have been attempted to minimise intestinal barrier damage during the weaning transition.^11,12^ At present, variations in the diet compositions and supplementation with bioactive compounds, like oligosaccharides, seem to be feasible options for ameliorating weaning-associated intestinal injury in post-weaned piglets.^13,14^

Alginate, the most abundant polysaccharide of brown algae, consists of β-d-mannuronic acid and α-l-guluronic acid as monomeric units,^15,16^ arranged as homopolymeric (poly-β-d-mannuronate (M-blocks) and poly-α-l-guluronate (G-blocks)) and heteropolymeric (MG-blocks) regions.^17,18^ Alginate oligosaccharide (AOS), a depolymerised product of alginate by alginate lyase, has attracted increasing attention, due to its diverse biological activities, such as anti-oxidation,^19^ anti-apoptotic,^20^ anti-inflammatory^21^ and anti-proliferative effects.^22^ In support of these properties, AOS could have applications in the food industry, as a functional dietary supplement for both humans and animals. Strikingly, a recent study demonstrated that AOS supplements had health-promoting effects on intestinal development in weaned pigs, particularly, enhancing the intestinal integrity.^23^ Nevertheless, to date, there is no specific information explaining the underlying mechanisms by which AOS improves the intestinal integrity of weaned pigs. Therefore, further studies regarding this intriguing topic are necessary.

Accordingly, the current study was undertaken to ascertain how dietary AOS inclusion beneficially influences the intestinal integrity in weaned pigs. Our findings will raise understanding about the role of AOS in preventing and treating intestinal barrier impairment in post-weaned piglets. These observations will also have important practical implications for developing AOS as a therapeutic agent to ameliorate intestinal barrier disruption in humans.

## Materials and methods

AOS, with an average molecular weight < 2000 Da, was obtained from the Dalian Institute of Chemical Physics, Chinese Academy of Sciences (Dalian, China).

### Animal care and experimental design

In a 14-day experiment, 24 crossbred pigs (Duroc × Landrace × Yorkshire), weaned at 21 days and with an average body weight of 6.21 (±0.09) kg, were assigned to two treatment groups (*n* = 12). One group was given a basal diet (control, CON) and the other group received the basal diet + 100 mg kg^−1^ AOS (termed the AOS group).

Table 1 displays the corn-soybean basal diet that was formulated to meet the nutrient requirements according to the National Research Council.^24^ All pigs were individually housed in 0.7 × 1.5 m metabolism cages in a temperature (24–26 °C) and humidity (60–70%) controlled nursery room. The diets were fed four times per day at 08 : 00, 12 : 00, 16 : 00 and 20 : 00 h, throughout the experiment, with *ad libitum* access to feed and water.

**Table 1:** Ingredients and nutrient composition of the basal diet

Ingredients	%	Nutrient composition^c^	%
Corn	28.80	Digestible energy (MJ kg^−1^)	14.85
Extruded corn	26.00	Crude protein	19.35
Soybean meal	11.00	Calcium	0.83
Extruded soybean	10.00	Total phosphorus	0.60
Whey powder	7.00	Available phosphorus	0.43
Soybean protein concentrate	5.00	Lysine	1.37
Fish meal	4.00	Methionine	0.49
Sucrose	4.00	Methionine + cysteine	0.76
Soybean oil	1.50	Threonine	0.81
Limestone	0.75	Tryptophan	0.22
Dicalcium phosphate	0.60		
l-Lysine·HCl (78%)	0.40		
NaCl	0.30		
dl-Methionine	0.18		
l-Threonine (98.5%)	0.10		
Chloride choline	0.10		
Tryptophan (98%)	0.02		
Vitamin premix^a^	0.05		
Mineral premix^b^	0.20		
Total	100		

a The vitamin premix provided the following per kg of diets: 6000 IU vitamin (V) A, 3000 IU VD_3_, 24 mg VE, 3 mg VK_3_, 1.5 mg VB_1_, 6 mg VB_2_, 3 mg VB_6_, 0.02 mg VB_12_, 14 mg niacin, 15 mg pantothenic acid, 1.2 mg folic acid and 0.15 mg biotin.

b The mineral premix provided the following per kg of diets: 100 mg Fe, 6 mg Cu, 100 mg Zn, 4 mg Mn, 0.30 mg I and 0.35 mg Se.

c Calculated composition.

### Sample collection

On day 15, before the morning feeding (08 : 00 h), eight pigs from each group were randomly selected for blood samples, collected *via* jugular vein puncture into non-anticoagulant vacuum tubes. Afterwards, the blood samples were centrifuged at 3500 × g, 4 °C, for 15 min, to separate the serum, and then stored at −20 °C.

After blood sample collection, the same pigs were euthanised by intravenous injection of sodium pentobarbital (200 mg kg^−1^ body weight). Afterwards, the abdomen was incised, and the small intestine was dissected free of the mesentery and arranged in measured lengths on a chilled stainless-steel tray. First, approximately 5 and 10 cm duodenal, jejunal and ileal segments were gently flushed with ice-cold phosphate buffered saline (PBS). Next, the 5 cm duodenal, jejunal and ileal segments were fixed in 4% paraformaldehyde solution and stored at 4 °C until toluidine blue staining, immunofluorescence and immunohistochemistry. The remaining 10 cm duodenal, jejunal and ileal segments were taken to collect mucosa samples by scraping with a scalpel blade, and then stored at −80 °C for quantitative real-time polymerase chain reaction (qPCR) and western blot analyses.

### Serum parameters assay

Serum cortisol, endotoxin, d-lactic acid, diamine oxidase (DAO) and corticotropin-releasing hormone (CRH) levels were measured, following the directions provided with the corresponding enzyme-linked immunosorbent assay (ELISA) kits (Beijing Winter Song Boye Biotechnology Co., Ltd., Beijing, China). All determinations were done in triplicate, and absorbance was measured using a multi-mode microplate reader (SpectraMax M2, Molecular Devices, Sunnyvale, CA, USA).

### Small intestine biochemical analysis

#### Tissue samples preparation

Frozen intestinal mucosa samples were rapidly thawed and then mixed with ice-cold physiological saline at a ratio of 1 : 9 (w/v). Next, the mixtures were centrifuged at 3000 × g, 4 °C, for 15 min, to acquire the supernatants. Before storage at −20 °C, the protein concentration in the mucosal homogenates supernatant was determined using the bicinchoninic acid method.^25^ The intestinal cytokines and antioxidant-related indices were standardised to the protein concentration in each sample.

#### Inflammatory cytokines measurements

Interleukin-1 (IL-1), IL-6, IL-10, tumour necrosis factor-α (TNF-α) and interferon-γ (IFN-γ) concentrations in the supernatant of mucosal homogenates were measured using the corresponding ELISA kits (Beijing Winter Song Boye Biotechnology Co., Ltd). The determinations were consistent with those used in measuring the serum factors, as described above.

#### Antioxidant status evaluation

Several antioxidant-related factors, including superoxide dismutase (SOD), catalase (CAT), glutathione (GSH), malondialdehyde (MDA) and total antioxidant capacity (T-AOC) were measured to evaluate the antioxidant status in the small intestine. All these determinations were made using the assay kits and associated protocols supplied by Nanjing Jiancheng Bioengineering Institute (Nanjing, China).

### Toluidine blue staining

For toluidine blue staining, the paraformaldehyde-fixed duodenal, jejunal and ileal tissues were embedded in paraffin and cut into 5 μm thick sections, dewaxed and hydrated.^26^ Next, the sections were stained in 0.5% toluidine blue at room temperature for 30 min, rinsed in water for 10 min and then differentiated in 95% ethanol. Lastly, the sections were dehydrated through absolute ethanol, cleared with xylene, sealed with neutral resin, and then observed under a Lecia DM1000 LED light microscope (Leica Microsystems, Wetzlar, Germany). On each slide, five different fields were counted, and the mast cell count per specimen was expressed as the mast cell count per mm^2^.

### Immunofluorescence

After 72 h paraformaldehyde fixation, the duodenal, jejunal and ileal samples for immunofluorescence were rinsed in PBS, transferred to 30% sucrose in PBS, infiltrated overnight, and were embedded the next day in O.C.T. compound (Sakura Finetek Co., Ltd., Tokyo, Japan) for frozen tissue specimens. Next, the duodenal, jejunal and ileal samples were cut into 5 μm thick sections, using a semi-automatic freezing microtome at −20 °C and mounted on glass slides. The sections were permeabilised with 0.5% Triton X-100 in PBS, at room temperature for 10 min. After washing three times with PBS, the sections were blocked with 10% goat serum in PBS at room temperature for 30 min, followed by incubation overnight at 4 °C with rabbit anti-occluding (at 1 : 100 dilution, Abcam plc., Cambridge, UK) antibody. After washing with PBS three times, the sections were incubated with a FITC-conjugated goat anti-rabbit IgG secondary antibody (Beijing Zhongshan Golden Bridge Biotechnology Co., Ltd., Beijing, China) at 37 °C for 30 min, followed by counterstaining with 4′,6-diamidino-2-phenylindole (DAPI) at room temperature for 10 min. Finally, after washing as described above, the sections were sealed with an anti-fluorescence quencher, and occluding protein distribution was visualised under a laser scanning confocal microscope (FV1000; Olympus Corporation, Tokyo, Japan).

### Immunohistochemistry

Immunohistochemistry was performed on duodenal, jejunal and ileal sections (2 μm thickness) that had been prepared as described above for toluidine blue staining. Briefly, the sections were pre-treated with 3% H_2_O_2_ in methanol at room temperature for 10 min to quench endogenous peroxidase activity and, then, heated in 10 mM citrate buffer (pH 6.0) for antigen retrieval. After washing with PBS three times, the sections were blocked with 10% goat serum at room temperature for 20 min to eliminate non-specific antibody binding. Subsequently, the sections were incubated overnight at 4 °C with 1 : 150 dilution rabbit anti-mast cell tryptase (PL Laboratories Inc., Newport Drive Port Moody, British Columbia, Canada) or 1 : 200 dilution rat anti-mast cell chymase (Abcam plc) antibodies. After washing with PBS three times, the sections were incubated with biotinylated goat anti-rabbit or anti-rat IgG secondary antibodies (Beijing Zhongshan Golden Bridge Biotechnology Co., Ltd) at 37 °C for 30 min. After washing with PBS three times, immunodetection was conducted, using 3,3′-diaminobenzidine (DAB) as the chromogen. The sections were then counterstained with haematoxylin and mounted in neutral resin. For each section in the Motic BA210 digital microscope (Motic China Group Co., Ltd., Xiamen, China), five fields of vision were randomly selected, with a fixed window area. The integrated optical density of tryptase- and chymase-positive mast cells in the duodenal, jejunal and ileal mucosa was detected by using Image-Pro Plus 6.0 image analysis system (Media Cybernetics, Bethesda, MD, USA). The mast cell tryptase and chymase expression levels were reflected by the mean value of the integrated optical density.

### Total RNA isolation and reverse transcription

Total RNA was extracted from frozen duodenal, jejunal or ileal samples (about 0.1 g), which were pulverised in liquid nitrogen and subsequently homogenised in 1 mL of RNAiso Plus (Takara Biotechnology Co., Ltd., Dalian, China), according to the manufacturer's instructions. The integrity and quality of total RNA were estimated by 1% agarose gel electrophoresis and the 260 nm/280 nm absorbance ratio (ideal ratio being within 1.8 and 2.0). Total RNA concentration in the final preparations was investigated at 260 nm using a spectrophotometer (NanoDrop 2000, Thermo Fisher Scientific Inc., Waltham, MA, USA). Afterwards, 1 μg total RNA of each duodenal, jejunal or ileal sample was used to synthesise cDNA, based on the protocol accompanying the PrimeScript™ RT reagent kit with gDNA Eraser (Takara Biotechnology Co., Ltd). The synthesis involved two steps: 37 °C for 15 min and 85 °C for 5 s.

### qPCR

Toll-like receptor 4 (TLR4), myeloid differentiation factor 88 (MyD88), interleukin-1 receptor-associated kinase 1 (IRAK1), tumour necrosis factor receptor-associated factor 6 (TRAF6), nucleotide-binding oligomerisation domain protein 1 (NOD1), nucleotide-binding oligomerisation domain protein 2 (NOD2) and receptor-interacting serine/threonine-protein kinase 2 (RIPK2) mRNA levels in intestinal mucosa were quantified using qPCR, as described by Wan *et al.*^27^ Briefly, the specific primers (Table 2) were designed using Primer Express 3.0 software (Applied Biosystems, Foster City, CA, USA) and purchased from Sangon Biotech Co., Ltd. All qPCR reactions were done in triplicate on a QuanStudio™ 6 Flex Real-Time PCR System (Applied Biosystems), using SYBR® Premix Ex Taq™ II (Tli RNaseH Plus; Takara Biotechnology Co., Ltd). Amplification was performed in a final volume of 10 μL. This solution consisted of 5 μL SYBR *Premix Ex* Taq II (Tli RNaseH Plus, 2×), 0.2 μL ROX Reference Dye II (50×), 0.4 μL forward primer (4 μM), 0.4 μL reverse primer (4 μM), 1 μL cDNA and 3 μL diethylpyrocarbonate-treated water. The following cycling conditions were applied: 95 °C for 30 s, followed by 40 cycles using a step program (95 °C for 5 s and 60 °C for 34 s). At the end of amplification, melting curve analysis was performed at 95 °C for 15 s, 60 °C for 1 min and 95 °C for 15 s, to confirm the specificity of the amplification reaction. Target and housekeeping gene amplification efficiencies were calculated according to the specific gene standard curves that were generated from 10-fold serial dilutions, quantifying six concentrations. After verification that the primers amplified with an efficiency close to 100%, the results were analysed using the 2^−ΔΔCt^ method,^27^ with porcine glyceraldehyde-3-phosphate dehydrogenase (GAPDH) gene as the housekeeping gene.

**Table 2:** Primer sequences for quantitative real-time polymerase chain reaction

Genes^a^	Primer sequence (5′–3′)	Size (bp)	Accession no.
TLR4	Forward: TCAGTTCTCACCTTCCTCCTG	166	GQ503242.1
	Reverse: GTTCATTCCTCACCCAGTCTTC		
MyD88	Forward: GATGGTAGCGGTTGTCTCTGAT	148	AB292176.1
	Reverse: GATGCTGGGGAACTCTTTCTTC		
IRAK1	Forward: CAAGGCAGGTCAGGTTTCGT	115	XM_003135490.1
	Reverse: TTCGTGGGGCGTGTAGTGT		
TRAF6	Forward: CAAGAGAATACCCAGTCGCACA	122	NM_001105286.1
	Reverse: ATCCGAGACAAAGGGGAAGAA		
NOD1	Forward: CTGTCGTCAACACCGATCCA	57	AB187219.1
	Reverse: CCAGTTGGTGACGCAGCTT		
NOD2	Forward: GAGCGCATCCTCTTAACTTTCG	66	AB195466.1
	Reverse: ACGCTCGTGATCCGTGAAC		
RIPK2	Forward: CAGTGTCCAGTAAATCGCAGTTG	206	XM_003355027.1
	Reverse: CAGGCTTCCGTCATCTGGTT		
GAPDH	Forward: ATGGTGAAGGTCGGAGTGAAC	235	NM_001206359.1
	Reverse: CTCGCTCCTGGAAGATGGT		

a TLR4, toll-like receptor 4; MyD88, myeloid differentiation factor 88; IRAK1, interleukin-1 receptor-associated kinase 1; TRAF6, tumour necrosis factor receptor-associated factor 6; NOD1, nucleotide-binding oligomerisation domain protein 1; NOD2, nucleotide-binding oligomerisation domain protein 2; RIPK2, receptor-interacting serine/threonine-protein kinase 2; GAPDH, glyceraldehyde-3-phosphate dehydrogenase.

### Western blot analysis

Approximately 0.1 g frozen intestinal mucosa samples were homogenised in 1 mL ice-cold RIPA lysis buffer (1% Triton X-100, 10% SDS, 0.15 M NaCl, 15.4 mM Tris–HCl, 0.5% deoxycholic acid, 1 μM Na orthovanadate, Roche Mini-EDTA-free Protease Inhibitor Cocktail; pH 8.0). The homogenate was centrifuged at 10 000 × g, 4 °C, for 10 min, and then the protein concentration in the supernatant was determined, as described above. Intestinal mucosa protein (40 μg) from each sample was mixed with 2× loading buffer and denatured by boiling at 95 °C for 5 min. After cooling, the samples were separated by 12% SDS-PAGE and transferred electrophoretically to polyvinylidene fluoride (PVDF) membranes (Bio-Rad Laboratories, Inc., Richmond, CA, USA).

The PVDF membranes were blocked (1 h at room temperature) in 5% non-fat dry milk in Tris-buffered saline containing 0.1% Tween-20 (TBS-T) before incubation with primary antibody. After thoroughly rinsing with TBS-T, the membranes were respectively incubated with rabbit anti-occluding (at 1 : 500 dilution, Abcam plc), rabbit anti-phospho-nuclear factor-κB (p-NF-κB) p65 (at 1 : 500 dilution, Cell Signalling Technology, Inc., Danvers, MA, USA) or rabbit anti-GAPDH (at 1 : 15 000 dilution, Abcam plc) antibodies, with gentle agitation overnight at 4 °C. Subsequently, the membranes were rinsed several times with TBS-T and then incubated with HRP-conjugated goat anti-rabbit IgG secondary antibody, at room temperature for 1 h (at 1 : 5000 dilution; Abcam plc). Finally, the membranes were rinsed several times with the same buffer at room temperature for 10 min each time. Blots were developed using a Clarity™ Western ECL Substrate (Bio-Rad Laboratories, Inc). The bands were visualised by exposure to X-OMAT BT films (Beyotime Institute of Biotechnology, Shanghai, China) for 1 min, and were quantified by using Quantity One software (Bio-Rad Laboratories, Inc). The relative abundance of each target protein was expressed as the ratio of targeted protein to GAPDH protein.

### Statistical analysis

All data analyses were performed using the Student's *t*-test in SAS 9.0 (SAS Inst., Inc., Cary, NC, USA), with each pig as an experimental unit. Values are expressed as mean ± standard error. The significance level was set at *P* < 0.05.

## Results

### Serum parameters

AOS supplementation decreased (*P* < 0.05) serum d-lactic acid content and DAO activity by 7.35% and 49.34%, respectively (Table 3). However, there was no difference (*P* > 0.05) in the serum cortisol, endotoxin and CRH levels between the two groups.

**Table 3:** Effects of alginate oligosaccharide on the serum parameters of weaned pigs^a^^b^

Items^d^	Treatments^c^		*P*-value
	CON	AOS	
Cortisol (ng mL^−1^)	145.55 ± 7.84	139.65 ± 6.15	0.563
Endotoxin (EU mL^−1^)	21.06 ± 0.56	20.42 ± 0.34	0.342
d-Lactic acid (μg mL^−1^)	26.67 ± 0.69	24.71 ± 0.53*	0.040
CRH (pg mL^−1^)	53.90 ± 1.19	50.40 ± 1.16	0.053
DAO (U L^−1^)	9.89 ± 0.65	5.01 ± 0.71**	<0.001

a *P < 0.05 *versus* the CON group. **P < 0.01 *versus* the CON group.

b Values are the means of 8 replicates per treatment.

c CON, a corn-soybean basal diet; AOS, alginate oligosaccharide (the basal diet supplemented with 100 mg kg^−1^ alginate oligosaccharide).

d CRH, corticotropin-releasing hormone; DAO, diamine oxidase.

### Intestinal antioxidant status

The differences in antioxidant-related variables in the small intestine between the two groups revealed AOS supplementation not only increased (*P* < 0.05) T-AOC but also decreased (*P* < 0.05) MDA content in the duodenum, jejunum and ileum (Table 4). Furthermore, compared to the control, a higher (*P* < 0.05) CAT activity was detected in the jejunum and ileum of the AOS group, but not (*P* > 0.05) in the duodenum. However, there were no significant changes (*P* > 0.05) in SOD activity and GSH content throughout the small intestine, between the two groups.

**Table 4:** Effects of alginate oligosaccharide on the intestinal antioxidant status of weaned pigs^a^^b^

Items^d^	Treatments^c^		*P*-value
	CON	AOS	
**Duodenum**			
SOD (U mg^−1^ protein)	19.27 ± 1.10	21.69 ± 0.99	0.125
CAT (U mg^−1^ protein)	5.08 ± 0.39	5.96 ± 0.27	0.083
GSH (mg g^−1^ protein)	10.36 ± 0.37	11.44 ± 0.86	0.278
MDA (nm mg^−1^ protein)	0.60 ± 0.04	0.36 ± 0.03**	<0.001
T-AOC (U mg^−1^ protein)	0.29 ± 0.03	0.42 ± 0.04*	0.033

**Jejunum**			
SOD (U mg^−1^ protein)	21.76 ± 0.79	23.41 ± 0.69	0.139
CAT (U mg^−1^ protein)	6.47 ± 0.42	7.87 ± 0.46*	0.042
GSH (mg g^−1^ protein)	9.99 ± 0.72	11.50 ± 0.78	0.178
MDA (nm mg^−1^ protein)	0.94 ± 0.07	0.60 ± 0.06**	0.003
T-AOC (U mg^−1^ protein)	0.54 ± 0.06	0.75 ± 0.07*	0.039

**Ileum**			
SOD (U mg^−1^ protein)	19.64 ± 1.20	21.52 ± 1.23	0.294
CAT (U mg^−1^ protein)	2.72 ± 0.15	4.67 ± 0.41**	0.002
GSH (mg g^−1^ protein)	6.36 ± 0.41	6.48 ± 0.26	0.817
MDA (nm mg^−1^ protein)	1.18 ± 0.08	0.77 ± 0.05**	<0.001
T-AOC (U mg^−1^ protein)	0.64 ± 0.06	1.01 ± 0.04**	<0.001

a *P < 0.05 *versus* the CON group. **P < 0.01 *versus* the CON group.

b Values are the means of 8 replicates per treatment.

c CON, a corn-soybean basal diet; AOS, alginate oligosaccharide (the basal diet supplemented with 100 mg kg^−1^ alginate oligosaccharide).

d SOD, superoxide dismutase; CAT, catalase; GSH, glutathione; MDA, malondialdehyde; T-AOC, total antioxidant capacity.

### Intestinal integrity

**Fig. 1:**
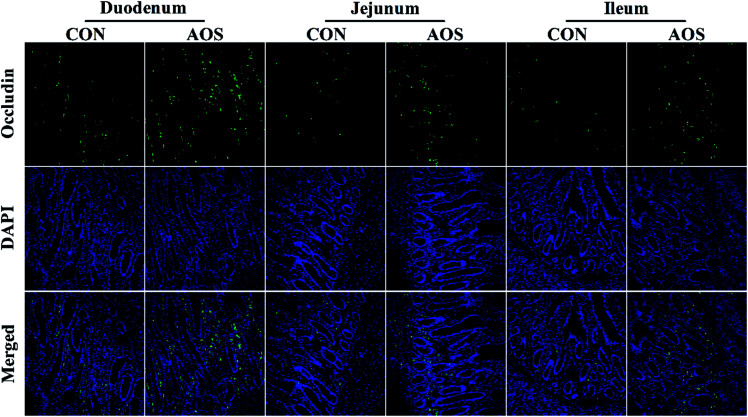
Occludin protein distribution in the small intestine of weaned pigs supplemented with or without alginate oligosaccharide (immunofluorescence;×400). CON, a corn-soybean basal diet; AOS, alginate oligosaccharide (the basal diet supplemented with 100 mg kg^−1^ alginate oligosaccharide). DAPI, 4′,6-diamidino-2-phenylindole.

**Fig. 2:**
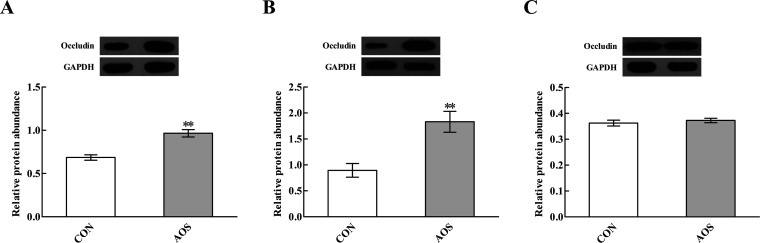
Effects of alginate oligosaccharide on the occludin protein abundance in the duodenum (A), jejunum (B) and ileum (C) of weaned pigs. Values are means (8 pigs/treatment), with standard errors represented by vertical bars. **P < 0.01 (indicates that the occludin protein abundance between the AOS group and the CON group differ significantly). CON, a corn-soybean basal diet; AOS, alginate oligosaccharide (the basal diet supplemented with 100 mg kg^−1^ alginate oligosaccharide). GAPDH, glyceraldehyde-3-phosphate dehydrogenase.

Immunofluorescence results indicated the occludin protein distribution in the small intestine was affected by AOS intervention (Fig. 1). For further confirmation, we determined the occludin protein abundance by western blot assay. AOS supplementation elevated (*P* < 0.05) the occludin protein abundance in the duodenum and jejunum, whereas this variable did not differ (*P* > 0.05) in the ileum (Fig. 2).

### Mast cell counts and phenotypes

**Fig. 3:**
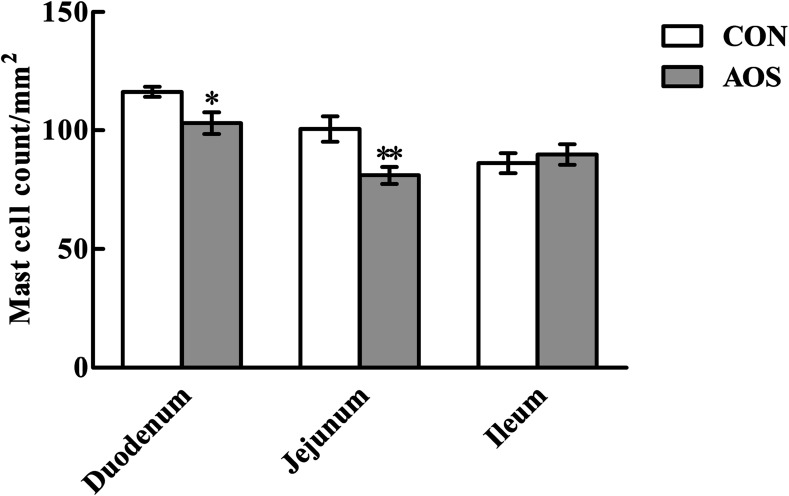
Effects of alginate oligosaccharide on the mast cell counts in the small intestine of weaned pigs (toluidine blue staining). Values are means (8 pigs/treatment), with standard errors represented by vertical bars. *P < 0.05 or **P < 0.01 (indicates that the mast cell counts between the AOS group and the CON group differ significantly). CON, a corn-soybean basal diet; AOS, alginate oligosaccharide (the basal diet supplemented with 100 mg kg^−1^ alginate oligosaccharide).

Fig. 3 shows a significant decrease (*P* < 0.05) in duodenal and jejunal mast cell counts in the AOS group compared to the CON group. However, ileal mast cell counts did not differ (*P* > 0.05) between the two groups.

**Fig. 4:**
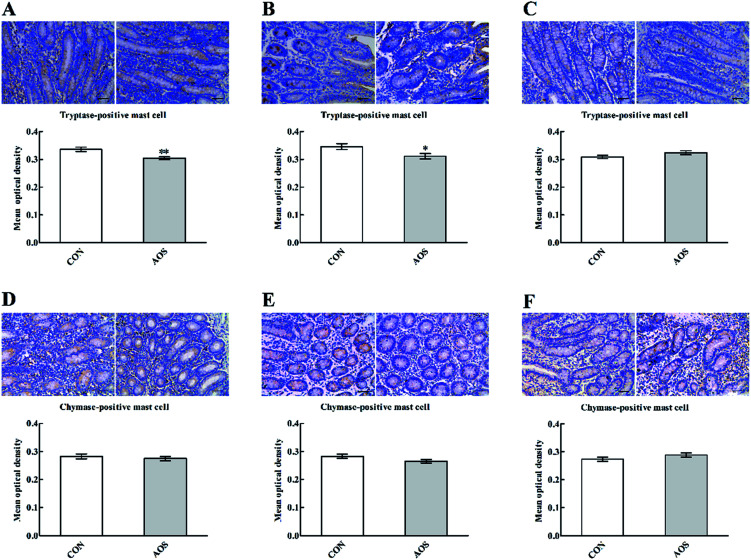
Effects of alginate oligosaccharide on the mast cell phenotypes in the duodenum (A and D), jejunum (B and E) and ileum (C and F) of weaned pigs (immunohistochemistry;×400). Values are means (8 pigs/treatment), with standard errors represented by vertical bars. *P < 0.05 or **P < 0.01 (indicates that the mast cell phenotypes between the AOS group and the CON group differ significantly). CON, a corn-soybean basal diet; AOS, alginate oligosaccharide (the basal diet supplemented with 100 mg kg^−1^ alginate oligosaccharide).

Although AOS did not significantly influence (*P* > 0.05) the mast cell tryptase expression in the ileum, the mast cell tryptase expressions in the duodenum and jejunum were significantly lower (*P* < 0.05) in the AOS-treated pigs than control pigs (Fig. 4). However, no variation (*P* > 0.05) regarding the mast cell chymase expression was found in the small intestine after dietary treatments.

### Intestinal cytokine contents

As depicted in Table 5, AOS supplementation only decreased (*P* < 0.05) TNF-α content in the duodenum and jejunum. Furthermore, dietary AOS inclusion increased (*P* < 0.05) IL-10 content without affecting (*P* > 0.05) IL-1, IL-6 and IFN-γ contents in the small intestine.

**Table 5:** Effects of alginate oligosaccharide on the intestinal cytokine contents of weaned pigs^a^^b^

Items^d^	Treatments^c^		*P*-value
	CON	AOS	
**Duodenum**			
IL-1 (pg mg^−1^ protein)	32.18 ± 0.80	30.46 ± 0.96	0.189
IL-6 (pg mg^−1^ protein)	117.48 ± 6.64	98.64 ± 6.01	0.054
IL-10 (pg mg^−1^ protein)	19.74 ± 1.13	27.49 ± 2.41*	0.012
TNF-α (pg mg^−1^ protein)	55.89 ± 2.81	46.22 ± 2.73*	0.027
IFN-γ (pg mg^−1^ protein)	79.26 ± 5.33	74.27 ± 5.21	0.514

**Jejunum**			
IL-1 (pg mg^−1^ protein)	35.73 ± 1.56	34.39 ± 1.72	0.572
IL-6 (pg mg^−1^ protein)	126.15 ± 8.48	113.91 ± 6.38	0.268
IL-10 (pg mg^−1^ protein)	18.10 ± 1.01	29.32 ± 2.26**	<0.001
TNF-α (pg mg^−1^ protein)	59.95 ± 3.36	49.92 ± 3.07*	0.045
IFN-γ (pg mg^−1^ protein)	85.25 ± 5.05	81.20 ± 4.15	0.545

**Ileum**			
IL-1 (pg mg^−1^ protein)	32.25 ± 1.66	29.55 ± 1.03	0.190
IL-6 (pg mg^−1^ protein)	95.28 ± 5.81	90.84 ± 5.79	0.597
IL-10 (pg mg^−1^ protein)	15.85 ± 1.32	29.97 ± 2.12**	<0.001
TNF-α (pg mg^−1^ protein)	41.80 ± 2.92	37.53 ± 2.37	0.275
IFN-γ (pg mg^−1^ protein)	70.55 ± 4.73	68.72 ± 3.43	0.758

a *P < 0.05 *versus* the CON group. **P < 0.01 *versus* the CON group.

b Values are the means of 8 replicates per treatment.

c CON, a corn-soybean basal diet; AOS, alginate oligosaccharide (the basal diet supplemented with 100 mg kg^−1^ alginate oligosaccharide).

d IL-1, interleukin-1; IL-6, interleukin-6; IL-10, interleukin-10; TNF-α, tumour necrosis factor-α; IFN-γ, interferon-γ.

### Expressions of TLR and NOD signalling-related genes

**Fig. 5:**
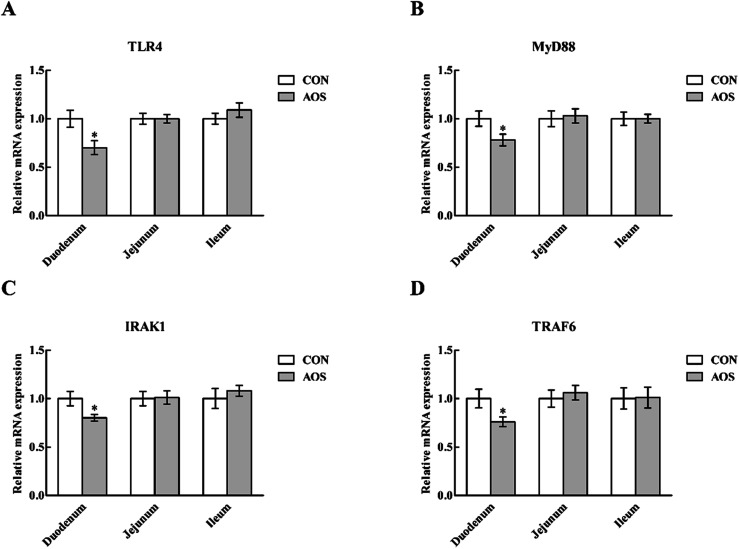
Relative mRNA levels of TLR4 (A), MyD88 (B), IRAK1 (C) and TRAF6 (D) in the small intestine of weaned pigs supplemented with or without alginate oligosaccharide. Values are means (8 pigs/treatment), with standard errors represented by vertical bars. *P < 0.05 (indicates that the mRNA expression of TLR signalling-related genes between the AOS group and the CON group differ significantly). CON, a corn-soybean basal diet; AOS, alginate oligosaccharide (the basal diet supplemented with 100 mg kg^−1^ alginate oligosaccharide). TLR4, toll-like receptor 4; MyD88, myeloid differentiation factor 88; IRAK1, interleukin-1 receptor-associated kinase 1; TRAF6, tumour necrosis factor receptor-associated factor 6.

**Fig. 6:**
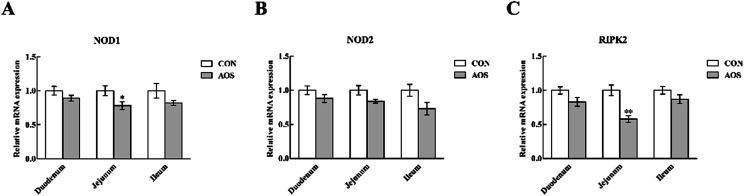
Relative mRNA levels of NOD1 (A), NOD2 (B) and RIPK2 (C) in the small intestine of weaned pigs supplemented with or without alginate oligosaccharide. Values are means (8 pigs/treatment), with standard errors represented by vertical bars. *P < 0.05 or **P < 0.01 (indicates that the NOD signalling-related genes between the AOS group and the CON group differ significantly). CON, a corn-soybean basal diet; AOS, alginate oligosaccharide (the basal diet supplemented with 100 mg kg^−1^ alginate oligosaccharide). NOD1, nucleotide-binding oligomerisation domain protein 1; NOD2, nucleotide-binding oligomerisation domain protein 2; RIPK2, receptor-interacting serine/threonine-protein kinase 2.

In the duodenum, TLR4, MyD88, IRAK1 and TRAF6 mRNA levels were lower (*P* < 0.05) in AOS-supplemented pigs than those in the control group (Fig. 5). AOS supplementation also decreased (*P* < 0.05) the jejunal NOD1 and RIPK2 mRNA levels (Fig. 6). However, ileal expressions of TLR and NOD signalling-related genes were not prominently influenced (*P* > 0.05) by supplemental AOS.

### p-NF-κB p65 protein abundance

**Fig. 7:**
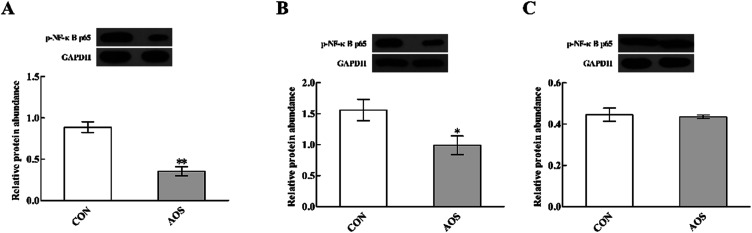
Effects of alginate oligosaccharide on the p-NF-κB p65 protein abundance in the duodenum (A), jejunum (B) and ileum (C) of weaned pigs. Values are means (8 pigs/treatment), with standard errors represented by vertical bars. *P < 0.05 or **P < 0.01 (indicates that the p-NF-κB p65 protein abundance between the AOS group and the CON group differ significantly). CON, a corn-soybean basal diet; AOS, alginate oligosaccharide (the basal diet supplemented with 100 mg kg^−1^ alginate oligosaccharide). p-NF-κB p65, phospho-nuclear factor-κB p65. GAPDH, glyceraldehyde-3-phosphate dehydrogenase.

Dietary supplementation with AOS resulted in down-regulation (*P* < 0.05) of p-NF-κB p65 protein abundance in the duodenum and jejunum (Fig. 7). However, the ileum was not affected (*P* > 0.05).

## Discussion

Apart from the function of the digestion and absorption of nutrients, the intestinal epithelium serves as a barrier against noxious antigens and pathogens.^29^ Therefore, maintaining an intact intestinal barrier is imperative to ensure adequate provision of dietary nutrients to the whole body and to prevent the penetration by luminal bacteria and dietary allergens into the mucosa.^30^ The epithelial junctional complex is a vital component of the intestinal barrier, and tight junctions are an important element of the epithelial junctional complex.^31,32^ The tight junctions are comprised of several unique proteins, such as occludin, an integral transmembrane protein with functional importance in maintaining the integrity of tight junctions.^33–35^ In this study, we observed that AOS up-regulated the occludin protein abundance in the duodenum and jejunum, indicating that AOS could enhance the intestinal integrity of weaned pigs. Serum d-lactic acid and DAO are two well-established markers for monitoring the changes in intestinal permeability; increases in d-lactic acid content and DAO activity in the serum are shown to correlate with the extent of intestinal barrier injury.^36,37^ Consistent with this perspective, we found a decreased serum d-lactic acid concentration and DAO activity following AOS supplementation, further supporting that AOS benefits the intestinal barrier integrity of weaned pigs.

An imbalance in the oxidative and antioxidant systems induces oxidative stress in the intestinal tissues of post-weaned piglets, which may disrupt the intestinal barrier integrity.^38,39^ It has been reported that marine-derived bioactive substances, such as AOS, may function as a powerful antioxidant to relieve oxidative stress in swine production.^40^ Here, the antioxidant status of the small intestine was evaluated by monitoring several antioxidant-related indices, including SOD, CAT, GSH, MDA and T-AOC. CAT is considered as the main enzyme responsible for eliminating free radicals (*e.g.*, hydroxyl radicals), and T-AOC indicates the protective capacity of the non-enzymatic antioxidant defence system.^41,42^ As shown, AOS supplementation not only increased CAT activity in the jejunum and ileum but also elevated T-AOC throughout the small intestine. These results suggest AOS could accelerate the intestinal antioxidant defence capacity in weaned pigs, through influencing both enzymatic and non-enzymatic antioxidants. Also, we found that AOS ingestion decreased MDA content in the small intestine, demonstrating that AOS could prevent the intestinal lipid peroxidation in weaned pigs.^43,44^ These combined results validated that AOS has a protective effect against oxidative stress resulting from weaning. Consequently, AOS supplementation partially contributed to preserving the intestinal barrier integrity of weaned pigs.

In addition to causing oxidative stress in the intestinal tissues of piglets, weaning also triggers intestinal inflammation in piglets, marked by an up-regulated expression of pro-inflammatory cytokines in the intestine.^45,46^ Most pro-inflammatory cytokines, such as TNF-α, induce a pathologic opening of the intestinal tight junction barrier, thereby increasing the intestinal epithelial permeability.^47–49^ Importantly, mast cells are the dominant cell type to store TNF-α and are thus rapidly primed for triggering TNF-α mediated inflammatory responses.^50,51^ In the present study, we found that AOS supplementation decreased the mast cell counts in the duodenum and jejunum. As expected, an accompanying decreased TNF-α concentration in the duodenum and jejunum was noticed, which is in agreement with the enhanced intestinal barrier integrity. Mast cells are also abundant in pre-formed granule mediators, such as tryptase and chymase, which, when released, have a profound influence on intestinal function, including increased intestinal permeability, inflammation and visceral hypersensitivity.^52,53^ The results presented demonstrated that AOS ingestion decreased duodenal and jejunal mast cell tryptase expression, signifying an amelioration of mast cell degranulation in the AOS-supplemented pigs. Collectively, these findings convey the notion that AOS suppressed both mast cell inflammatory mediator production and degranulation, thereby preventing weaning-associated intestinal inflammation in piglets. Based on the current scientific evidence, inflammatory responses can be regulated by a variety of signalling pathways.^54,55^ Thus, we also investigated the molecular mechanisms by which AOS affects the intestinal inflammatory responses in weaned pigs.

TLRs are an ancient conserved family of pattern-recognition receptors that play a critical role in recognising microbial pathogens and modulating antimicrobial host defence.^56^ TLR4 is the best-characterised member of this family and is activated by endotoxin or lipopolysaccharide (LPS) from Gram-negative bacteria and initiates the systemic inflammatory response syndrome.^57,58^ Here, we discovered that duodenal TLR4 mRNA level and its down-stream signals, including MyD88, IRAK1 and TRAF6, were decreased in AOS-treated pigs. Therefore, the improved intestinal integrity after AOS intervention may be closely related to the suppressed production of the intestinal pro-inflammatory cytokines *via* inhibition of the TLR4 signalling pathway. Besides TLRs, other pattern recognition receptors, like cytoplasmic NOD proteins have a critical role in recognising pathogen-associated molecular patterns and regulating the innate immune responses.^59^ NODs bind with the bacterial LPS and peptidoglycan, activating a TLR-independent signal, which also results in NF-κB activation *via* RIPK2 and stimulates the expression of pro-inflammatory cytokines.^60^ Interestingly, similar to our observations regarding the TLR4 signalling pathway, we discovered that jejunal NOD1 mRNA level and its adaptor molecule RIPK2 were down-regulated in AOS-supplemented pigs. Thus, the enhanced intestinal integrity following AOS supplementation was also possibly related to reducing the intestinal pro-inflammatory cytokines production *via* inhibition of the NOD1 signalling pathway. Next, we confirmed that AOS supplementation down-regulated the p-NF-κB p65 protein abundance in the duodenum and jejunum. Thus, we concluded that the decrease in the AOS-mediated intestinal pro-inflammatory cytokines synthesis in weaned pigs was directly linked to blocking NF-κB expression *via* suppression of the TLR4 and NOD1 signalling pathways.

## Conclusions

AOS supplementation exerted beneficial effects in improving the intestinal integrity of weaned pigs. This behaviour was closely related to the enhanced antioxidant capacity and decreased mast cell degranulation, as well as prevention of mast cell pro-inflammatory cytokines release, *via* restraining the TLR4/NF-κB and NOD1/NF-κB signalling pathways.

## Conflicts of interest

The authors declare that they have no competing interests.

## Ethics approval

All animal care protocols in this study were performed in accordance with the Animal Management Rules of the Ministry of Health of the People's Republic of China and approved by the Animal Care and Use Committee of Sichuan Agricultural University (Chengdu, China).

## Supplementary Material
